# Advancing STI priorities in the SDG era: priorities for action

**DOI:** 10.1186/s12992-018-0331-3

**Published:** 2018-01-16

**Authors:** Matthew F. Chersich, Sinead Delany-Moretlwe, Greg Martin, Helen Rees

**Affiliations:** 1 Wits Reproductive Health and HIV Institute, Faculty of Health Sciences, University of the Witwatersrand, 1 Esselen Street Hillbrow, Johannesburg, 2000 South Africa; 2 Health Protection Surveillance Centre, Dublin, Republic of Ireland

**Keywords:** Sexually transmitted infections, Sexually transmitted diseases, Sustainable development goals

## Abstract

The Sustainable Development Goals present an opportunity to reimagine and then reconfigure the approach to controlling sexually transmitted infections (STIs). The predilection of STIs for women and for vulnerable populations means that services that ameliorate STIs, by their nature, enhance equity, a key focus of the goals. Given the considerable breadth and depth of the goals, it is important to locate points of convergence between the SDGs and STIs, further craft synergies with HIV and select a few population groups and settings to prioritise. There are many opportunities for STI aficionados in this era to advance the field and global control of these infections.

## Where do STIs fit within the SDGs?

The far-reaching, highly ambitious Sustainable Development Goals (SDGs) build upon the momentum generated by the Millennium Development Goals (MDGs), and will guide health, social and economic initiatives until 2030 [1]. As with its predecessor, the SDGs are likely to influence the allocation of resources for global health programmes. Issues marginal to the SDGs are likely to receive limited national and global attention, even if these issues are significant in themselves. Within the MDG period, for example, spending by bilateral agencies on non- communicable diseases was actually higher in the 1990s than in the late 2000s, when the MDGs were well-established. Thus, fields that align more closely with the SDGs can expect to receive a considerable boost in this era, while those that are not are likely to suffer.

SDG 3, the overarching goal specific to health, seeks to ensure healthy lives and promote wellbeing for all, across the lifespan. The goal addresses a wider range of conditions than the health-related MDGs. Target 3.3, for example, extends beyond the three diseases central to the MDGs (HIV, tuberculosis and malaria), and encompasses neglected tropical diseases, along with the combating of hepatitis, water-borne diseases, and other communicable diseases. Aside from breadth, there is also greater depth to SDG 3 compared to its MDG counterparts. SDG 3 expressly acknowledges that health cannot be attained without social and economic changes.

At first glance, however, the place given to health appears somewhat diminished in the SDGs, where it constitutes only 1 of 17 goals, while in the MDGS it formed 3 of 8. This observation might hold true for health in general, but sexual and reproductive health, by contrast, has gained in prominence [2]. There is indeed much cause for optimism. Sexual and reproductive health and rights are included as specific targets under both the health (SDG 3) and gender (SDG 5) goals, and these, in turn, are explicitly linked to the International Conference on Population and Development (ICPD) and Beijing Platforms; conferences that articulated reproductive rights for women and the services they require to achieve these (See Table 1). Notably, achieving gender equity is a standalone goal in the SDGs, a major achievement for global gender advocates. Clearly, progress in this domain would lessen women’s vulnerability to STIs, gender related violence and the health consequences of fewer economic and social opportunities, all of which are inter-linked. The SDG framework is, however, not all good news for STI aficionados. There is no direct mention of the term ‘STIs’ within the SDGs, while several other communicable diseases are specifically named. Also missing is direct reference to adolescents’ rights to SRH services and information, including comprehensive sexuality education, although these were strongly advocated for in the long lead-up to the SDG declaration.

**Table 1:** SDG targets relevant to STIs and opportunities for maximising the impact of STI services in this new era

Target	SDG target^a^	Opportunities for maximising STI impact on SDG
**Goal 3: Ensure healthy lives and promote well-being for all at all ages**		
**3.1**	By 2030 end **preventable deaths of newborns** and under-five children	***EMTCT of syphilis*** STI interventions in maternal health services, pint of care diagnostics
**3.3**	By 2030 **end the epidemics of AIDS**, tuberculosis, malaria, and neglected tropical diseases and **combat hepatitis**, water-borne diseases, and **other communicable diseases**	EMTCT of syphilis, HBV vaccine, combat other STIs STI interventions in maternal health services, POC diagnostics
**3.5**	Strengthen **prevention and treatment of substance abuse**, including narcotic drug abuse and harmful use of alcohol	Combat effects of narcotic drugs and alcohol on sexual behaviour and thus STIs STI interventions in substance abuse programmes. STI behavioural research
**3.7**	By 2030 ensure **universal access to SRH services**, including for family planning, information and education, and the **integration of reproductive health into national strategies and programmes**	Universal access to STI services Integration of STIs within national strategies, including for HIV, adolescents and maternal health
**3.8**	**Achieve UHC**, including **financial risk protection**, access to quality essential health care services, and access to safe, effective, quality, and **affordable essential medicines and vaccines for all**	Universal access to STI services, and safe, effective, quality and affordable medicines (e.g. HPV) Access to STI vaccines Health financing: reduce private sector out-of-pocket payments; cash transfers for raising demand for services
**3.c**	Increase substantially health financing and the recruitment, **development and training and retention of the health workforce** in developing countries	**Raise capacity of health workers** in STI services
**3,d**	Strengthen the capacity of all countries, particularly developing countries, for early warning, risk reduction, and management of national and global health risks	National and global responses to STI epidemics or STI drug resistance
**Goal 5: Achieve gender equality and empower all women and girls**		
5.2	**Eliminate all forms of violence against all women and girls** …….including trafficking and sexual and other types of exploitation	**Reduce STI risks from sexual violence**, link services for STIs with those for sexual and gender-based violence
5.6	**Ensure universal access to SRH and reproductive rights as agreed in accordance with the Programme of Action of the ICPD and the Beijing Platform for Action**	STI services situated within **rights-based approach**, Women’s Empowerment, ICPD Programme of Action
**Goal 10: Reduce inequality within and among countries**		
10.3	Ensure equal opportunity and **reduce inequalities of outcome**, including through **eliminating discriminatory laws, policies and practices** and promoting appropriate legislation, policies and actions in this regard	STI services reduce inequalities by **targeting higher risk groups**, e.g. sex workers, young women and adolescents and men who have sex with men.
**Goal 17. Strengthen the means of implementation and revitalize the global partnership for sustainable development**		
17.6	Enhance North-South, South-South and **triangular regional and international cooperation on and access to science, technology and innovation**, and enhance knowledge sharing on mutually agreed terms, including through improved coordination among existing mechanisms, particularly at UN level, and through a global technology facilitation mechanism when agreed	**Advances in science, technology and innovation for STIs (e.g. vaccines, multi-purpose technologies)** North-South and South-South research
17.16	Enhance **the global partnership for sustainable development** complemented by multi-stakeholder partnerships that mobilize and share knowledge, expertise, technologies and financial resources	Further develop multi-stakeholder global partnerships for STIs Align, in detail, global STI strategies with SDGs, and foster a place for STIs within other global initiatives and commitments

Key opportunities and text of targets in bold. ^a^Sustainable Development Solutions Network: Indicators and a Monitoring Framework for the Sustainable Development Goals Launching a data revolution for the SDGs. 2015

Two other distinctive features of the SDGs bear mention. Firstly, the goals were purposively designed to be cross-cutting, forming a ‘network of targets’ [3], wherein each target explicitly refers to multiple goals [4]. The aim is a coherent approach spanning several sectors, rather than a set of isolated, silo-like activities. We argue that nowhere is the potential benefit of this interconnectedness clearer than in the area of STI control. Taken to its logical conclusion, the conditions under which STIs flourish would be mitigated by advances in ensuring quality healthcare and education in general (SDG 3 and 4); achieving gender equality (SDG 5); reductions in inequality and stigma within and between countries (SDG 10); promoting economic growth and decent work (SDG 8); making cities safe and resilient (SDG 11); and the creation of global partnerships for research and sustainable development (SDG 17).

A second defining feature is that the goals are applicable to all countries, regardless of income levels. Coherent broad initiatives that have global relevance, especially those that interrogate and counter inequalities, are a major underpinning of the SDGs. This aims to challenge the *modus operandi* of policy makers and researchers alike. Unlike many other diseases, STIs are a major contributor to the disease burden in all countries and have relatively similar epidemiological profiles across economic and geographical areas. Potentially, then, it is possible to develop one cohesive global STI control strategy that sets out the priority interventions for each specific STI and the cluster of indicators that can be applied universally to all countries, regardless of economic status.

In short, the SDGs present an opportunity to reimagine and then reconfigure global health, and to accentuate their centrality to sustainable development. The global health community, however, is yet to fully take on board the implications of ‘sustainable development’, and how this is conceptually distinct from ‘poverty reduction’, the main MDG objective. The kind of sustainable and inclusive development envisaged by the SDGs demands that those working in fields such as STIs reconceptualise anew the health services that are needed now. Failure to do so risks relegating the SGDs to the pile of failed health initiatives past [5].

## Framework for advancing STI control within the SDGs

The remainder of this paper proposes a framework for refocusing attention on the control of STIs in the forthcoming years, most especially within low- and middle-income Countries (LMICs). Given the considerable breadth and depth of the SDGs, is not difficult to locate points of convergence between the SDGs and STIs. STI services hold many competitive advantages in this new era. The danger might even be trying to align the STI agenda with too many of the SDG targets, rather than framing STIs within a few carefully-selected areas. Furthermore, a moulding of STI approaches to the SDGs needs to be cognisant that the MDG period was a challenging time for the STI field. Disease prevalence and especially drug resistance rose alarmingly in many settings. Major gaps in service delivery and monitoring persisted or even worsened. Moreover, STIs were given little attention within the large global initiatives that characterised the MDG period, such as The Global Fund and the Bill and Melinda Gates Foundation. Even large commissions on health investments included only the HPV vaccine among the ‘Best buy clinical interventions’ [6], ignoring other value-for-money interventions for STIs. The framework suggested below is therefore shaped both by opportunities within the SDG period, but also by the constraints carried over from the recent past. Broadly, we propose 1. the strategic promotion of a few effective and emerging STI interventions; 2. further crafting of synergies with HIV; and 3. the selection of a few population groups and settings to prioritise. The text largely pertains to STIs other than HIV; considerations specific to HIV and the SDGs are presented elsewhere [7].

### Strategically promote selected STI interventions

It is critical that aspects of STIs highlighted within national and global policy documents (such as the WHO Global Health Sector Strategy on STIs 2016–2021) [8], reinforce a program of work fully in tune with the SDGs, and use consistent messaging throughout. This messaging will also be useful as Global Health Initiatives continue to progressively align themselves with the SDGs. This alignment will present opportunities for the STI field to garner more attention and to build on the momentum stemming from success of the Global elimination of Mother to Child Transmission of HIV (eMTCT), HIV and Syphilis efforts, and of the marked technological advances in the field of STI diagnostics. A clearly defined program of work could galvanise these actions and expand the place of STIs within Global Initiatives. Practically, in order to do so, the specific steps required to attain the SDG targets pertinent to STI need to be defined.

The ambitious aims of the SDGs call for disease paradigms to be remoulded, incorporating emerging and neglected interventions, and specifically be directed at inequities and integrative approaches. Emergent multi-purpose technologies and point-of-care tests will alter the face of STI care. Periodic presumptive treatment for groups such as sex workers is a good example of a neglected intervention [9]. The HPV vaccine is already considered an intervention of high global importance and could spearhead STI control progress. An HSV vaccine would also spur the field on – the WHO Product Development for Vaccines Advisory Committee lists this vaccine among the top 10 priority vaccines to be developed [10].

In fact, there is good evidence on most of the steps needed to ‘end/eliminate STIs public health impact by 2030’, and doubtless new exciting interventions will emerge. Together these must capture global position, imagination and funding spaces. Overall, placing access to STI services within the ambit of Universal Health Care frameworks (Target 3.8), a key underpinning of the SDGs, might gain more traction than simple calls for ‘improved’ STI services. Lastly, as with all communicable diseases, the STI field needs to demonstrate its responsiveness to epidemics (SDG 3,d), both those related to transmission of infections, and of drug resistant microbes.

The opportunity for a fresh start gives the field a chance to move on from any previous failures in research or programming, and to renew its focus on things that will work well. Noting programme and research successes and being clear on how to propagate them will provide areas to be championed, which the SDG era is crying out for. This era is seeking compelling approaches where success can be demonstrated – the highlighting of solutions, rather than the demonstration of need or gaps in services. Several notable examples of success already exist. For instance, with a comprehensive program, syphilis declined in nearly all higher-risk groups in China [11–13] and substantial overall reductions were noted globally. The Cuba HIV syphilis eMTCT achievements mark another case in point [14]. Appreciable declines in incidence of chancroid, syphilis and gonococcal rates, in sequelae such as neonatal conjunctivitis, and in access to HPV vaccination in high-income countries (HICs), are clearly championable causes.

### Further mend the ‘fractured paradigm’ between HIV and STI

The convergence of HIV and syphilis within the eMTCT program was a major achievement during the MDGs, and the integrated nature of these efforts neatly reflects the core principles of the SDGs. The SDG era provides the perfect foil for mending the ‘fractured paradigm’ [15], one in which STI ‘fundamentals’ were ignored and rules reinvented for HIV. This paradigm has been as counterproductive for HIV as it has been for non-HIV STIs. In many countries, during the MDG period, basic STI services were left in disarray as STI program resources dwindled, and major HIV epidemics emerged from and then spread rapidly under conditions of poor STI control. Any further weakening of STI control may well undermine progress made in HIV prevention. Fundamentally, wider STI control *is* feasible and HIV prevention can be strengthened in doing so.

There are many examples that illustrate the potential synergies between HIV and the STI field, essentially inseparable areas. The eMTCT HIV and syphilis example has already been cited. The Global AIDS Response Progress Reporting (GARPR) [16] now includes STI indicators to inform HIV and STI prevention and control interventions at local level and to monitor progress of national programmes. Prior to GARPR, congenital syphilis reporting, for example, was not routinely collected at global level. Well-developed HIV services, such as ART clinics, provide similar platforms for collaboration. Further initiatives aiming for convergence between HIV and other STIs could form central pillars of STI approaches to the SDGs.

### Focus on vulnerable groups and on equity gains

The predilection of STIs for women and for vulnerable populations makes these infections intrinsically inequitable. Services that ameliorate STIs, by their nature, thus enhance equity. By contrast, NCD interventions, for example, often address comparatively better off groups, primarily older adults and elderly, and can actually widen gaps in health outcomes [17]. Framing STI services as a key vehicle to reducing health and other inequities is potentially of much strategic value.

Some population groups are central to both the STI field and the attainment of the SDGs. Young women (15–24 years) in both LMICs and high-income countries, in particular, are arguably the highest priority group for STI control, and are given centre-place within many facets of the SDGs. The growing attention and funding for this group could well be channelled into combatting the complex multi-sectoral factors underpinning their heightened vulnerability, including to STIs. STI services in Africa warrant attention (Table 2), especially the targeting of young women. Migrant populations also garner attention in the less health focused SDGs and are important for STI control. Presenting STI interventions as an important means of attaining youth and migrant goals, for example, might well gain some traction.

**Table 2:** Prioritise poorly performing areas, especially Africa

• Compelling SDG-centred arguments can be made for prioritising resources for STI services in Africa
• Only 14 of 47 sub-Saharan African countries completed WHO STI surveillance survey, only 10 of which have routine STI surveillance [18]
• Many success stories of STIs in Sri Lanka, syphilis control in Cuba, and sex worker programmes in India and elsewhere, but few or no programme-level STI success examples are from Africa, though much empirical research occurs here!
• Compared to large donor and government-driven sex work projects in Asia, almost all similar programmes in Africa are small isolated projects, research focused and have limited coverage. The effectiveness of sex work services that are comprehensive, as opposed to health-only, directly reflects the principles of the SGDs.

Addressing vulnerability includes lowering the financial barriers to service access. Many intersections between health financing and STIs remain unexploited, especially demand side incentives. Conditional cash transfers, for example, for partner notification and for attendance of STI screening and treatment among groups such as sex workers or men who have sex with men, tick many of the SDG boxes. Also, rather than being perceived as an obstacle to STI progress, the disproportionate role played by the private sector in treatment of STIs presents opportunities for public-private sector linkages, again consistent with the SDG framework.

## Conclusions

The STI field as a whole needs to develop a response to the question: ‘Given the nature of the SDGs, how do we go about justifying investment and accelerating progress in STIs in this new climate?’. Business as usual is unlikely to work, it had mixed success in MDG era, and might even do worse under the SDGs. There is a considerable risk of getting lost in the ocean of competing priorities. STI global directives, research agenda and especially services will need to be carefully aligned with the case made. Like what was done in other fields, a Global Commission involving low-, middle- and high-income countries, potential funding agencies, STI experts and policy leads could examine the opportunities and priorities for STIs in the next 15 years, and present a focused program of work. Also, practically, future STI conferences could be themed around SDGs, contributing to the development of catchphrases such as: “Ending STIs impact on public health by 2030”. These Commissions, conferences and catchphrases could shape the vision and strategies around STI in this era.

Done right, the STI agenda may well fit better with the SDGs than MDGs. The necessarily integrative, complex nature of STI control better suits the SDGs, than the more vertical responses promoted by the MDGs. Each disease area needs to pro-actively make their case in these early years of the SDGs, noting how they contribute to other disease areas and to several SDG targets. Paradoxically, it might be best to strongly promote only a select few STI interventions and vulnerable populations that are integrative, have multiple impacts and can champion STIs role in attaining the SDGs. The alternative, presenting arguments for comprehensive packages of clinical services, risks getting lost in the relative complexity of the SDGs.

Sustainable development prerogatives have already fomented global shifts (in understandings of climate change, for example). Can the SDGs also usher in a golden era for STIs? Quite possibly, an end to STIs as a public health problem is possible by 2030, with a pro-active, focused agenda crafted around a few compelling interventions relevant to all countries, synergised with HIV and targeted at vulnerable populations; all framed within the SDGs.

## Abbreviations

LMICs: Low- and middle-income countries.
MDGs: Millennium Development Goals.
SDGs: Sustainable Development Goals.
STIs: Sexually transmitted infections.
